# Insights into Cis-Amide-Modified Carbon Nanotubes for Selective Purification of CH_4_ and H_2_ from Gas Mixtures: A Comparative DFT Study

**DOI:** 10.3390/ma17143588

**Published:** 2024-07-20

**Authors:** Atyeh Rahmanzadeh, Nasser AL-Hamdani, Evangelos P. Favvas, Giorgio De Luca

**Affiliations:** 1 Dipartimento di Fisica, Università Della Calabria, 87036 Rende, Italy; atyeh.rahmanzadeh@unical.it; 2 Institute on Membrane Technology, ITM-CNR, Ponte P. Bucci, Cubo 17/c, 87036 Rende, Italy; n.alhamdani@itm.cnr.it; 3 Institute of Nanoscience and Nanotechnology, National Center for Scientific Research “Demokritos”, Aghia Paraskevi, 153 41 Athens, Greece; e.favvas@inn.demokritos.gr

**Keywords:** functionalized CNT, novel functional group, gas separation, quantum mechanics modeling, H_2_/CH_4_ mixtures purification

## Abstract

Among a plethora of mixtures, the methane (CH_4_) and hydrogen (H_2_) mixture has garnered considerable attention for multiple reasons, especially in the framework of energy production and industrial processes as well as ecological considerations. Despite the fact that the CH_4_/H_2_ mixture performs many critical tasks, the presence of other gases, such as carbon dioxide, sulfur compounds like H_2_S, and water vapor, leads to many undesirable consequences. Thus purification of this mixture from these gases assumes considerable relevance. In the current research, first-principle calculations in the frame of density functional theory are carried out to propose a new functional group for vertically aligned carbon nanotubes (VA-CNTs) interacting preferentially with polar molecules rather than CH_4_ and H_2_ in order to obtain a more efficient methane and hydrogen separations The binding energies associated with the interactions between several chemical groups and target gases were calculated first, and then a functional group formed by a modified ethylene glycol and acetyl amide was selected. This functional group was attached to the CNT edge with an appropriate diameter, and hence the binding energies with the target gases and steric hindrance were evaluated. The binding energy of the most polar molecule (H_2_O) was found to be more than six times higher than that of H_2_, indicating a significant enhancement of the nanotube tip’s affinity toward polar gases. Thus, this functionalization is beneficial for enhancing the capability of highly packed functionalized VA-CNT membranes to purify CH_4_/H_2_ gas mixtures.

## 1. Introduction

Over recent decades, interest in gas mixtures has surged due to their broad scientific and industrial applications. Gas mixtures are crucial in numerous industrial processes, such as optimizing chemical reactions and enhancing safety in manufacturing procedures [1,2,3,4,5], or as alternative energy sources because the gas composition directly affects combustion emissions and efficiency [6,7,8,9].

Among various mixtures, methane (CH_4_) and hydrogen (H_2_) have gained significant attention for their roles in energy production (hydrogen/methane mixtures produce cleaner fuel than methane alone), industrial processes, and environmental considerations [10,11,12,13]. Moreover, this mixture is pivotal in producing synthesis gas (syngas) [14,15,16] and can be used for renewable energy storage, where excess renewable energy generates hydrogen which is then mixed with methane for future use [17,18,19,20], supporting the transition toward more sustainable energy systems [21,22,23]. In industries, methane/hydrogen mixtures are used to synthesize chemicals and are crucial in sectors like metal processing, ammonia production, and petrochemicals [24,25,26,27,28].

However, the presence of other gases like carbon dioxide (CO_2_), ethane, propane, butanes, sulfur compounds (e.g., H_2_S), and water vapor (H_2_O) can lead to undesirable effects, such as reduced energy output, altered flame characteristics, corrosive impacts on pipelines and equipment, and decreased process efficiency [29,30,31]. Hence, purifying CH_4_/H_2_ mixtures, especially from CO_2_, H_2_S, and water vapor, is critical. In natural gas (NG) and biogas (BG) upgrading, removing CO_2_, H_2_S, and water vapor is essential, since these non-combustible components lower the calorific value and make transporting raw gas mixtures unprofitable [32,33]. Effective separation technologies with low carbon footprints are needed, particularly for pre-combustion CO_2_ capture from high-pressure NG and low-pressure BG streams.

Several techniques have been developed to separate CH_4_/H_2_ mixtures from other gases, including cryogenic distillation, absorption and adsorption, pressure swing adsorption (PSA), and membrane-based separation. Among these, membrane-based separation stands out due to its versatility, low operating costs, environmental benefits, continuous operation, compact design, and high energy efficiency [34,35,36]. In general, membrane selectivity depends on differences in molecular shape and size or the feed molecules’ affinity to the membrane [37,38]. Recently, nanostructured membranes have attracted attention due to their enhanced selectivity, increased permeability, stability, tailored functionality, and reduced thickness [39,40,41,42,43,44]. Nanostructured membranes offer precise control over the pore size and surface characteristics. Moreover, materials like carbon nanotubes (CNTs) or graphene facilitate gas transport, improving separation efficiency and reducing processing times [45,46,47]. They also exhibit advanced chemical and mechanical stability, which are essential for industrial applications, and can be designed with specific surface interactions and chemistries for selective separation. Their ultra-thin design facilitates fast mass transfer and reduces the overall size of separation systems [48,49].

Incorporating CNTs into mixed matrix membranes can significantly improve gas permeability [50,51]. Aligned nanotubes, especially when oriented vertically (VA-CNTs) with respect to the separating membrane’s layer, dramatically increase permeability, making them a special nanostructure for membrane-based separations [52,53]. CNTs exhibit ultrafast transport of low molecular weight gases, far surpassing common polymers in terms of gas permeation [54] (from 1 to 2 orders of magnitude). For example, Favvas et al. [50,51] reported that the He permeance increased from 18 to 107 GPU through the addition of surface-modified MWCNTs (4% *w*/*w* concentration) into a polyimide membrane matrix. This change was from 1.5 to ~37 GPU in the case of N_2_. Similar behavior was also reported by other groups [55,56]. VA-CNT arrays and ultrathin VA-CNT membranes can be fabricated using methods like chemical vapor deposition or solution-based techniques, where nanotubes are aligned and placed in a polymer matrix for stability [57]. In VA-CNT membranes, the functionalized CNTs determine membrane selectivity and permeability, depending on the CNT concentration and specific affinity for feed molecules [57,58,59,60,61,62]. However, achieving high selectivity remains challenging, and hence functionalizing CNTs with groups that preferentially interact with polar gases can enhance the purification of CH_4_/H_2_ mixtures containing CO_2_, H_2_S, and H_2_O.

Extensive research has focused on using nanostructured membranes for separating CH_4_/H_2_ and other gas mixtures, employing various experimental and computational methods [63,64,65]. Mixed-matrix membranes incorporating functionalized nanomaterials have drawn significant interest [65]. Hydrogen separation is a top priority for scientists and governments due to its role as a green energy carrier [66]. Mahboubian et al. used molecular dynamics (MD) simulations to evaluate a graphenylene membrane with a functionalized nanopore for CH_4_/H_2_ separation, revealing high H_2_ selectivity and no detectable CH_4_ permeation [67]. Zhu et al. predicted the potential of graphenylene membranes for gas separation using computer simulations, showing promise for new gas separation technologies [68].

Rudkevich’s review on the supramolecular chemistry of gases highlighted the interactions between secondary cis-amides and gases like N_2_O and CO_2_ through hydrogen-bonding and electrostatic interactions [69]. Ab initio calculations and molecular modeling studies confirmed hydrogen bond formation between cis-amides and gases, suggesting potential for gas separation applications [70].

**Scheme 1:**
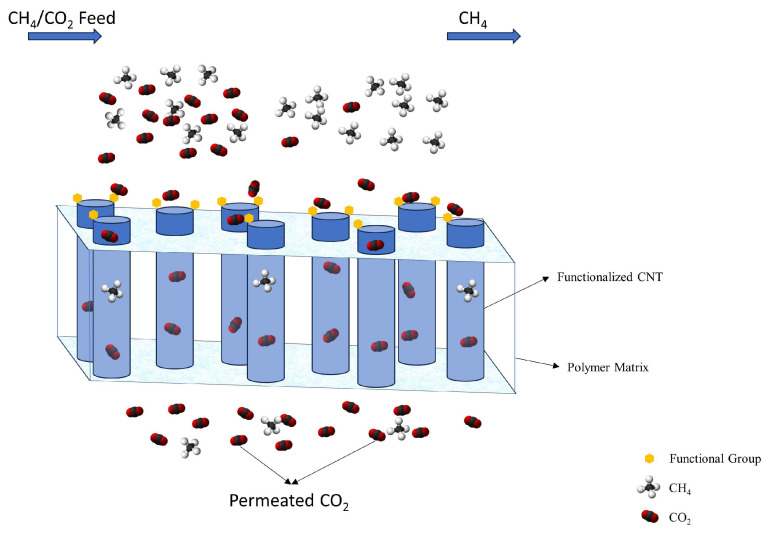
General illustration of a VA-CNT nanostructured membrane which inspired the calculations.

In the current research, first-principle calculations are carried out in the framework of density functional theory to scrutinize the affinity of functionalized VA-CNTs toward different polar gases, such as CO_2_, H_2_O, and H_2_S, and nonpolar gases, like CH_4_ and H_2_. In this regard, inspired by the two-point noncovalent interaction which secondary cis-amides or acetates can form with the above molecules, several chemical groups were investigated. Acetyl amide was chosen among the analyzed groups, and then a functional group (FG) formed by a modified ethylene glycol monomer was attached to the edge of a CNT with an appropriate diameter. The binding energies associated with the interactions between this functional group and the target gases were calculated to verify whether the high interaction between the proposed FG and the polar molecules is still retained when the former is anchored on the nanotube inlet. Scheme 1 illustrates in a general way a VA-CNT membrane, which inspired the calculations of this work.

The polymer among the VA-CNTs should show low affinity for the studied molecules and consist of low-cost materials so that gas permselectivity is carried out by the functionalized nanotubes.

## 2. Models and Computational Details

**Figure 1:**
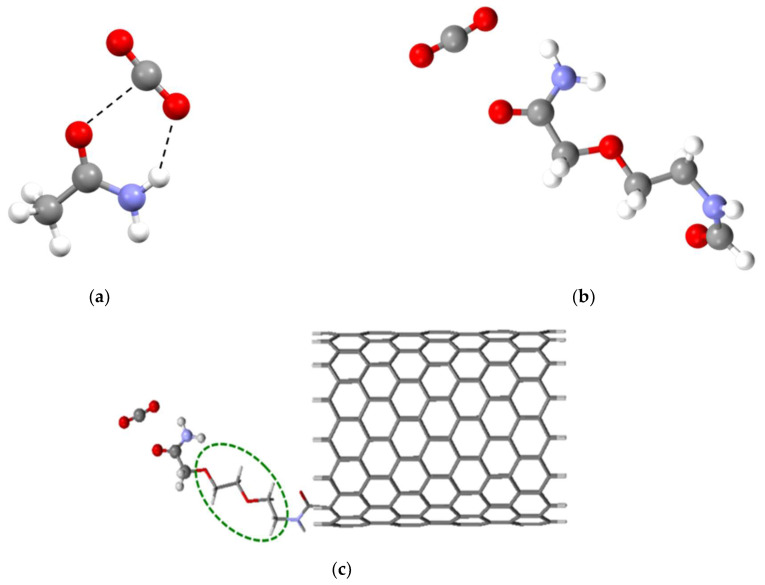
(**a**) A cis-amide based head group with a cis configuration. (**b**) A functional group composed of an ethylene glycol monomer and cis-amide head. (**c**) A functionalized CNT edge with the functional group (FG) highlighted within the green circle. Oxygen atoms are red, hydrogen atoms are white, carbon atoms are gray, and nitrogen atoms are light blue.

Functionalization of VA-CNTs is carried out using functional groups composed of spacers (hooks) and heads, as shown in Figure 1. Ethylene glycol oligomers (OEGs) have been successfully used to modify surface properties [71,72], and thus the chemistry for surface functionalization using OEGs is well known. In this work, modified OEGs are used to link specific chemical groups (i.e., the heads of FGs) to the inlets of CNTs.

The heads of the functional group (see Figure 1a and the top of Figure 1b) were inspired by Rudkevich’s review on the supramolecular chemistry of gases [69], which describes a two-point noncovalent interaction between the head and the gas molecules present. The configuration for this interaction, which drives selective adsorption of the polar gases, is shown in Figure 1a. It is worth mentioning that the electron donor and acceptor atoms in the heads must exhibit a cis configuration, and all of the head groups analyzed in this work present such a configuration.

The FG hooks have a terminal amino group, shown at the bottom of Figure 1b, which allows them to link the entire functional group to the CNT edge via an amide bond. This bond guarantees a certain stability for the functionalized CNT by preventing hydrolysis and resisting fairly high temperatures. The diameter of the CNT was calibrated according to the steric hindrance of the functional groups in order to allow access of the gaseous molecules into the CNT.

Quantum calculations were performed for closed-shell configurations using the Northwest Computational Chemistry Package (NWChem 6.6) [73] and the B3LYP exchange-correlation functional [74] (i.e., within the framework of the generalized gradient approximation). The coulomb and exchange-correlation potentials were numerically integrated into an adaptive grid with an accuracy of 10^−5^. In the calculations involving small structures, like heads and functional groups, the threshold for the energy convergence and the root mean square of the electron density were set to 10^−6^ (a.u.) and 10^−5^ (a.u.), respectively, while in the functionalized CNT calculations, both thresholds were set to 10^−5^ (a.u.). No smear or level shift values were imposed. The necessary dispersion corrections were considered in the DFT calculations according to Grimme’s DFT-D4 approach. Both the DFT-D3 and D4 quantum parameterizations of the total energy functional provide reliable results for carbon-based structures without excessive computational costs [75,76].

Geometry optimizations were carried out using the driver module implemented in NWChem and by adopting the default thresholds for the maximum and root mean square gradients, as well as for the maximum and root mean atom displacements. Furthermore, in the case of CO_2_, the Hessian calculation was carried out in order to verify whether the head-gas adducts were true minima on the potential energy surface by evaluating the internal vibration frequencies.

Different linear combinations of Gaussian-type orbitals were used in the calculations. In detail, a triple-ζ basis set plus a polarization function (6-311G*) was used for all atoms of the head-CO_2_ adducts, for which the internal vibration frequencies were also calculated. The same basis set was used for optimization of the FGs-CO_2_ geometries. After the preliminary screening of the head groups and hooks, we used a double-ζ orbital basis set (6-31G) on all atoms in the geometry optimizations of the selected FG interacting with H_2_O, H_2_S, CO_2_, CH_4_, and H_2_. Finally, due to the high computation times, we used the double-ζ orbital basis set for optimization of the functionalized CNT interacting with the above gas molecules.

In the calculation of the binding energy (BE), each adduct was separated into two subunits, namely the head, FG, or CNT-FG and the target gas molecules Then, the BE was calculated as the difference between the sum of the energies of the individual fragments (head-molecule, FG-molecule, or CNT-FG-molecule) and the energy of the adduct. Furthermore, the BEs were computed considering the basis set superposition error by using the counterpoise method [77].

## 3. Results and Discussion

### 3.1. Design of the Functional Group

First, a preliminary screening of the chemical groups was carried out in order to select the best head to use in a modified OEG by defining a reliable FG to be anchored on the CNT. To evaluate the ability of different chemical groups to form an effective two-point noncovalent interaction, we calculated their binding energies with carbon dioxide. This preliminary analysis allowed us to choose the best head group.

Table 1 shows the binding energies referring to 13 head groups with different pairs of atoms which formed a two-point noncovalent interaction. As pointed out, the calculation of the internal vibration frequency was performed for each of these head-CO_2_ complexes. All vibrational frequencies were positive, and therefore the obtained geometries were minima on the potential energy surface. The values reported in Table 1 show that the acetyl amide group provided the highest BE, confirming the conclusions and evidence reported in Rudkevich’s review [69]. In comparison with the different chemical groups, it is evident that a cis-amide arrangement involving C=0 and N-H moieties was the best configuration for an efficient interaction with carbon dioxide. It is worth noting that the ketone and acetate groups provided low-to-medium binding energies. In Table S1 (Supplementary Materials), the binding energies for the interactions of the 13 head groups with CO_2_ were calculated by using the X3LYP [78] functional to verify whether the same trend, calculated using the B3LYP functional, was found. The values in Table S1 show that once again, the acetyl amide group provided the highest BE, while the ketone and acetate groups provided low-to-medium binding energies. Ab initio calculations performed on the complexes formed by CO_2_ and carbonyl compounds [70] showed a binding energy of approximately 2.69 kcal/mol. This value is in good agreement with the binding energies found in this work for ketone, acetate, and acetaldehyde.

Furthermore, the Raman spectra of the complexes formed by acetaldehyde and CO_2_ showed the maximum redshift of the acetaldehyde carbonyl band to be from 1746.0 to 1743.5 cm^−1^ and the maximum blueshift of the aldehyde CH proton to be from 2717.0 to 2718.3 cm^−1^ [70]. Both observations are consistent with a two-point noncovalent interaction, as the computer-predicted structures indicated.

Based on these preliminary calculations, the acetate and acetyl amide heads, providing low and high binding energies, were chosen and attached to three OEGs formed by one, two, and three ethylene glycol monomers. The BEs, associated with the interaction with CO_2_, were calculated, and they are reported in Table 2. These values show that the OEG-acetyl amide once again provided the highest binding energies. In addition, the OEGs attached to the head groups did not significantly modify the BE of the head groups, as the values remained substantially unchanged compared to those in Table 1. Thus, the acetate head group still exhibited lower binding energies. In Table S2, the binding energies calculated using the X3LYP and referring to the interactions of the OEG-acetyl amide and acetate are reported. From the values in Table 2, it can be observed that the OEG-acetyl amide functional groups still provided a higher binding energy than the OEG-acetate, and the number of monomers did not affect the values of the binding energies.

This analysis highlights that the OEG-acetyl amide functional group can be used to functionalize the edges of CNTs and obtain nanotubes with high affinity toward polar gases, such as CO_2_.

### 3.2. Affinity of the Functionalized CNT Edge toward the Target Gases

Before proceeding with the analysis of the affinity of the functionalized CNT toward the investigated gases, we calculated the binding energies associated with the interactions between the acetyl amide head and the target gases. The calculated values are reported in Table 3 and Table 4, respectively. It is worth mentioning that the number of ethylene glycol monomers did not affect the binding energies of the heads, as shown in Table 2, and therefore a single monomer was used as the hook in the functional group. The wisdom of this choice will be discussed later. Importantly, the influence of the number of ethylene glycol monomers on the binding energies was tested for a maximum of three monomers. Further calculations, requiring molecular dynamics approaches, would be needed to understand the influence of the hook length on the interaction with gas molecules. This is beyond the scope of this work, as it would require evaluation of the steric hindrance of functional groups at the nanotube entrance.

The values reported in Table 3 show that the selected acetyl amide head group gave the highest BE with H_2_O molecules. This is plausible since water is the most polar molecule among those analyzed. Therefore, it could form stronger hydrogen bonds between the H_2_O oxygen and the hydrogen of the N-H group, as well as between the hydrogen of the water and the amide carbonyl, according to the arrangement shown in Figure 1a. Similar interactions were formed by H_2_S but to a lesser extent, since sulfur is less electronegative than water’s oxygen. Carbon dioxide continued to form a two-point noncovalent interaction with the amide group, but a slight difference in the binding energy, with respect to the value reported in Table 1, was found. This was due to the threshold criteria used in geometry optimization which, in this case, were less accurate than the stringent ones used in the previous optimization, in addition to the different basis set used in the last calculation. The nonpolar CH_4_ and H_2_ molecules showed comparable binding energies and markedly lower values compared with the polar gases. We found that the BE of H_2_O was about 14 times higher than that of H_2_ (larger by an order of magnitude), while the BE of CO_2_ was six times higher than the binding energy of the hydrogen molecule. Meanwhile, the binding energies were related to the adsorption of the gas molecules on the FG. This result is quite interesting, because it shows that the selected acetyl amide group allowed efficient selective adsorption of polar molecules with respect to methane and hydrogen, suggesting efficient selectivity of the functionalized CNTs.

The binding energies associated with the two-point interactions between the modified ethylene glycol-acetyl amide and target molecules are shown in Table 4. From this table, it can still be observed that the number of ethylene glycol monomers did not significantly modify the BEs. In general, all the binding energies decreased negligibly compared with the values reported in Table 3, and thus the trend found was still maintained, with the H_2_O BE being 14 times larger than that of H_2_. As a result, we can conclude that this functional group can be used to functionalize the edges of CNTs in order to significantly increase the affinity and selectivity of the nanotube toward polar gas molecules.

It should be noted that the number of ethylene glycol monomers used in the hook of the functional group should be calibrated according to the CNT diameter. Specifically, in order to obtain high selectivity for the CNTs toward small gaseous molecules, nanotubes with large diameters require various FGs on their edges with a greater number of monomers. Additionally, the orientation of the anchored FGs will be important in this case. FGs with a large number of monomers oriented toward the center of CNTs can hinder the diffusion of the gaseous molecules into the CNTs. In this regard, a good compromise between CNT-FG models and the computational time should also be considered, as CNT-FG models with large nanotube diameters require high computational times. Thus, we believe that for the proof of concept proposed in this work, a CNT with a diameter of 1.58 nm and a functional group formed by one modified ethylene glycol monomer in the hook is a good compromise.

**Figure 2:**
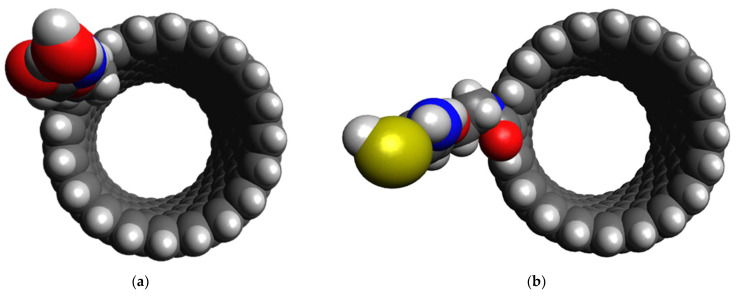

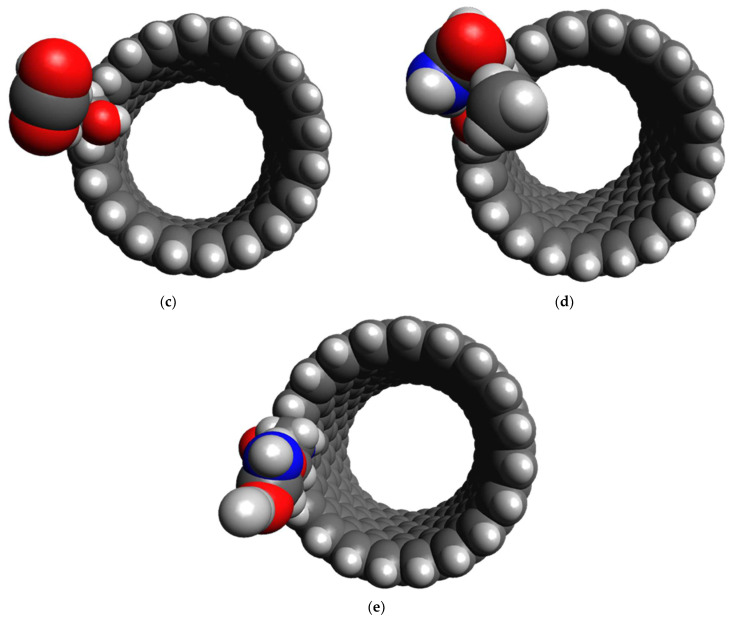
Top view of the functionalized CNT with the optimized structures of the selected functional group, interacting with (**a**) H_2_O, (**b**) H_2_S, (**c**) CO_2_ (**d**) CH_4_, and (**e**) H_2_. Oxygen atoms are red, hydrogen atoms are white, carbon atoms are gray, sulfur atoms are yellow, and nitrogen atoms are blue.

The optimized geometries of the CNT-FG (FG = 1EG-CH_2_CONH_2_) interacting with the target molecules are shown in Figure 2. The various optimized structures were visualized with Avogadro software (version 1.2.0) [79] by considering the van der Wall spheres on each atom of the systems in order to qualitatively evaluate the steric hindrance of the FG attached on the CNT. The structures show that the orientation of the functional group interacting with the target molecules was almost perpendicular to the inlet plane of the nanotube, except for the functional group, which interacted with the H_2_S molecules (Figure 2b). In this case, the optimized FG-H_2_S adduct was directed outside the CNT entrance, and this suggests that once the H_2_S is adsorbed, it is less likely to jump into the CNTs compared with the other gases.

**Figure 3:**
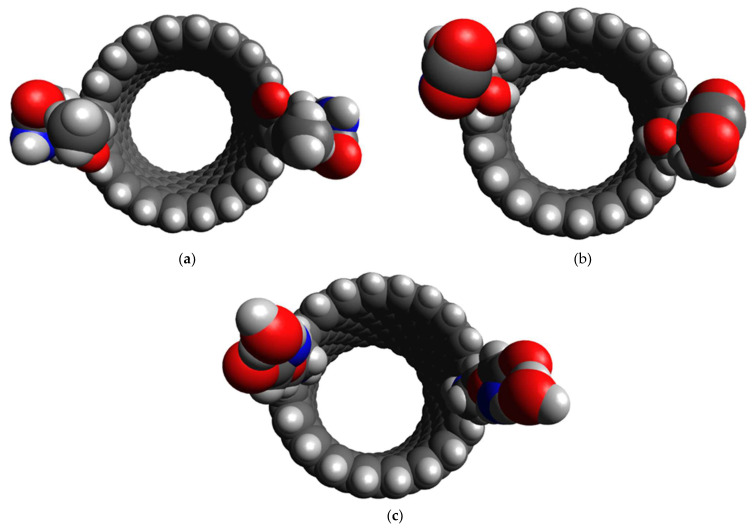
Top view of the functionalized CNT edge with two optimized functional groups, interacting with (**a**) H_2_O, (**b**) CO_2_, and (**c**) CH_4_. Oxygen atoms are red, hydrogen atoms are white, carbon atoms are gray, and nitrogen atoms are light blue.

However, these optimized structures demonstrate that the proposed FG attached to the CNT edge with a diameter of 1.58 nm did not hinder the diffusion of the target molecules inside the nanotube. Furthermore, the optimized structures of the CNT functionalized with two functional groups interacting with H_2_O, CO_2_, and CH_4_ are shown in Figure 3.

As for the CNT functionalized with a single functional group, the structures in Figure 3 again show that two functional groups on the edge of the nanotube did not inhibit the entrance of gaseous molecules into the CNT and therefore their diffusion. Thus, this topological analysis suggests that functionalization of the CNT with the proposed functional group, formed by the modified ethylene glycol and acetyl amide, will be beneficial in improving the affinity of the nanotube toward polar gas molecules. It is worth mentioning that the functional groups not interacting with the gas molecules might form an intermolecular interaction with each other at the inlet of the CNT. However, by using only one monomer of ethylene glycol, this interaction is less likely. As highlighted, the influence of the hook length was beyond the scope of this work, as it would require evaluation of the steric hindrance and intermolecular interaction between the functional groups at the nanotube entrance. Starting from the obtained outcomes, we calculated the binding energies, reported in Table 5, associated with the interactions between the functionalized CNT and the target molecules.

The values of Table 5 show a systematic increase in the binding energies. In particular, the BEs of the most polar gases (H_2_O and H_2_S) increased more than the energies of methane and hydrogen. The H_2_O BE in this case was more than six times higher than the binding energy of H_2_. Hence, when the proposed functional group was attached to the carbon nanotube, the ratio between the binding energies of H_2_O (the most polar gas) and that of H_2_ (less polar) was significantly reduced. Nevertheless, it remained significantly high. It is worth mentioning that the binding energies shown in Table 5 were associated with weak intermolecular interactions, and thus it is plausible that polar gases adsorbed on the functional groups can pass into the CNTs without requiring excessive energy to break the interaction with the FGs. Considering that the affinity of the nanotube inlet for a gas molecule is related to the corresponding binding energy linked with the interactions between the anchored functional group and the gas molecule, the values in Table 5 show that functionalization of nanotubes with the modified ethylene glycol-acetyl amide group would be beneficial to increasing the permselectivity of VA-CNT membranes toward polar gases.

There are some papers in the literature where CNTs (SWCNTs or MWCNTs) have been grafted using ethylene glycol-based groups but not the proposed acetyl amide [80,81,82,83]. Furthermore, there are not theoretical studies about how these groups on CNTs’ surfaces can affect the adsorption and diffusion in the literature yet, concluding in their separation. Thus, in this work, we analyzed the ability of some chemical groups to form two-point noncovalent interactions with light gases, in which an electrostatic interaction is coupled with a hydrogen bond. The group which provided the greatest two-point noncovalent interaction was therefore identified (i.e., acetyl amide). This represents a novel finding in the field of selective adsorption and functionalized supports. The polarity of the gas is a parameter controlling the selective adsorption because, as demonstrate in this study, the binding of each gas on the FG is correlated with the gas polarity. This aspect has not yet been explored in detail, and thus this work can be considered a reference (pioneer) for the design of other functional groups, providing the selective adsorption of polar gases through a two-point interaction mechanism which promotes greater diffusion of the molecules inside CNTs.

One final aspect which deserves to be mentioned is the analysis of the chemical reaction barriers. We are aware that the modeling of transition states is quite interesting when a real mixture is considered in the separation process for, above all, biogas upgrades, where a mixture of gases of CO_2_, H_2_S, O_2_, CH_4_, and H_2_O exists in the feed stream. Indeed, in this kind of mixture, reactions like H_2_S + O_2_ → H_2_O + SO_2_ and H_2_S + H_2_O → H_2_SO_4_ + H_2_, which conclude in H_2_SO_4_, SO_2_, and H_2_ formation, must be taken into account. However, before considering chemical reactions, when competing with molecular adsorption, the gas’s noncovalent interaction between the CNT-FG and target gases should be thoroughly evaluated to provide reliable functionalized support. The aim of this work was to verify, from a computational point of view, whether specific functional groups can interact selectively with polar gases compared with non-polar ones using a two-point non-covalent interaction. In particular, we wanted to suggest a specific FG for which the above interaction is maximal. Therefore, this research should be considered a preliminary study to other investigations, such as transition state analysis which goes beyond the scope of this work. It is worth noting that the general conclusions presented in this work, concerning the selective adsorption on the proposed FG, can be extended to molecules such as H_2_SO_4_, and SO_2_ as well.

## 4. Conclusions

In the current research, first-principle calculations were carried out in the framework of density functional theory to propose a new functional group for highly packed functionalized VA-CNT membranes which interact preferentially with the polar molecules CO_2_, H_2_O, and H_2_S rather than CH_4_ and H_2_. From a preliminary screening, modified ethylene glycol and acetyl amide functional groups were suggested.

The results show that the inlet of the carbon nanotube functionalized with the proposed functional group showed a greater affinity toward polar molecules compared with non-polar ones, the binding energy of H_2_O with the FG was more than six times higher than the binding energy of H_2_. This ratio is significantly high, suggesting that the functionalization of CNTs with a modified ethylene glycol-acetyl amide can be useful for increasing the permselectivity of nanotubes. Furthermore, from a topological analysis, it can be observed that the proposed FG did not exhibit high steric hindrance to the diffusion of the adsorbed gas molecules, even when considering two FGs per edge for the CNTs. This result predicts that polar gases adsorbed on a functional group can move into the CNT without requiring excessive energy while also considering that the correlated binding energies are associated with weak intermolecular interactions.

Bearing in mind that the affinity of functionalized surfaces to gas molecules is related to the corresponding binding energies, which are associated with the interactions between the functional group and molecules, this work suggests that CNT functionalization with the proposed functional group is beneficial for increasing the permselectivity of VA-CNT membranes toward polar gases, which is advantageous for several processes where separations of polar gases from nonpolar gases is required, such as biogas upgrading.

## Figures and Tables

**Table 1:** Binding energies, referring to the interaction between the head group and CO_2_.

Head Group	Binding Energy (kcal/mol)
CH_3_CSCH_3_	2.44
CH_3_COCH_3_	3.14
CH_3_COH	3.30
CH_3_OCOCH_3_	3.47
CH_3_OCOSH	3.75
HOCH_2_CH_2_NH_2_	4.29
CH_3_CSOH	4.63
CH_3_CSNH_2_	4.65
CH_3_SOONH_2_	5.05
HCONH_2_	5.06
CH_3_COOH	5.10
CH_3_OCONH_2_	5.29
CH_3_CONH_2_	5.34

**Table 2:** Binding energies, referring to the interaction between the OEG-head group and CO_2_. Acetate and acetyl amide were used as heads, while one, two, and three ethylene glycol monomers were considered to be the hooks.

Functional Group	Binding Energy (kcal/mol)
1 ethylene glycol-CH_2_OCOCH_3_	−3.54
2 ethylene glycol-CH_2_OCOCH_3_	−3.57
3 ethylene glycol-CH_2_OCOCH_3_	−3.57
1 ethylene glycol-CH_2_CONH_2_	−5.65
2 ethylene glycol-CH_2_CONH_2_	−5.64
3 ethylene glycol-CH_2_CONH_2_	−5.65

**Table 3:** Binding energies, referring to the interaction between the acetyl amide head group and H_2_O, H_2_S, CO_2_, CH_4_, and H_2_.

Molecule	Binding Energy (kcal/mol)
H_2_O	15.17
H_2_S	8.00
CO_2_	6.94
CH_4_	1.73
H_2_	1.10

**Table 4:** Binding energies, referring to the interaction between the 1EG-CH_2_CONH_2_ functional group and H_2_O, H_2_S, CO_2_, CH_4_, and H_2_.

Molecule	Binding Energy (kcal/mol)
H_2_O	14.84
H_2_S	7.85
CO_2_	6.84
CH_4_	1.46
H_2_	1.08

**Table 5:** Binding energies, referring to the interaction between CNT-FG and H_2_O, H_2_S, CO_2_, CH_4_, and H_2_ molecules.

Molecule	Binding Energy (kcal/mol)
H_2_O	17.67
H_2_S	11.84
CO_2_	8.11
CH_4_	2.90
H_2_	2.82

## Data Availability

The original contributions presented in the study are included in the article/Supplementary Materials, further inquiries can be directed to the corresponding author.

## Supplementary Materials

The following supporting information can be downloaded at https://www.mdpi.com/article/10.3390/ma17143588/s1, Table S1: Binding energies referring to the interaction between heads group and CO_2_ by using X3LYP functional; Table S2: Binding energies referring to the interaction between OEG-head group and CO_2_. Acetate and acetyl amide are used as heads while one, two and three ethylene glycol monomers were considered as the hooks.
