# Supplementary material for: Insights into Cis-Amide-Modified Carbon Nanotubes for Selective Purification of CH4 and H2 from Gas Mixtures: A Comparative DFT Study
Source: Materials (Basel). 2024 Jul 20;17(14):3588. doi: 10.3390/ma17143588 (PMC11279001; doi:10.3390/ma17143588)
Supplement: Supplementary file 1 [file materials-17-03588-s001.zip › materials-3050229-supplementary.pdf]

Supplementary Materials

Insights into Cis-Amide-Modified Carbon Nanotubes for Selective Purification of CH4 and H2 from Gas Mixtures: A Comparative DFT Study

Atyeh Rahmanzadeh[1], Nasser AL-Hamdani[2], Evangelos P. Favvas[3], Giorgio De Luca[2*]

[1]*Dipartimento di Fisica, University of Calabria, 87036 Rende CS, Italy*

[2]*Institute on Membrane Technology, ITM-CNR, Ponte P. Bucci, cubo 17/c, 87036 Rende, Italy*

[3] *Materials & Membranes for Environmental Separations Laboratory, Institute of Nanoscience and Nanotechnology National Center for Scientific Research "Demokritos", Aghia Paraskevi 153 41, Athens Greece*

Table S1 shows the binding energies calculated using X3LYP functional and associated to the two-point interaction between the chemical groups used as head of the functional group and carbon dioxide.

Table S1. Binding energies referring to the interaction between heads group and CO_2 by using X3LYP functional.

**Head Group**	**Binding Energy (Kcal/mol)**
CH_3CSCH_3	1.42
CH_3COCH_3	2.14
CH3COH	2.22
CH_3OCOCH_3	2.28
$HOCH_2CH_2NH_2$	2.49
CH3OCOSH	2.52
CH_3CSOH	3.38
CH_3CSNH_2	3.39
CH_3SOONH_2	3.62
$HCONH_2$	3.67
CH_3COOH	3.79
CH_3OCONH_2	3.83
CH_3CONH_2	3.92

The binding energies evaluated by using X3LYP functional and related to the intermolecular interaction between OEG-head group and CO_2 are reported in Table S2.

Table S2. Binding energies referring to the interaction between OEG-head group and CO_2. Acetate and acetyl amide are used as heads while one, two and three ethylene glycol monomers were considered as the hooks.

**Functional group**	**Binding Energy (kcal/mol)**
ethylene glycol $-CH_2OCOCH_3$	2.21
di-ethylene glycol $-CH_2OCOCH_3$	2.26
tri-ethylene glycol $-CH_2OCOCH_3$	2.25
ethylene glycol $-CH_2CONH_2$	4.18
di- ethylene glycol $-CH_2CONH_2$	4.17
tri- ethylene glycol $-CH_2CONH_2$	4.18
